# To Disguise the Taste of Medicines

**Published:** 1883-08

**Authors:** 


To Disguise the Taste of Medicines.—Bitter and nauseous salines are best taken simply diluted with iced water. A mouthful or two of iced water, before or after the dose, to blunt the sense of taste, and the dose between them in a wineglassful of iced water, renders it easily taken by most persons.—*Squibb's Ephemeris.*

